# The roles of TNFAIP2 in cancers and infectious diseases

**DOI:** 10.1111/jcmm.13822

**Published:** 2018-08-25

**Authors:** Lin Jia, Yundong Shi, Yi Wen, Wei Li, Jing Feng, Ceshi Chen

**Affiliations:** ^1^ Department of Biology Yuxi Normal University Yuxi China; ^2^ Key Laboratory of Animal Models and Human Disease Mechanisms of the Chinese Academy of Sciences and Yunnan Province Kunming Institute of Zoology Chinese Academy of Sciences Kunming China; ^3^ Department of Urology of the First People's Hospital of Yunnan Province Kunming China; ^4^ Medical College of Kunming University of Science and Technology Kunming China; ^5^ Department of Laboratory Medicine & Central Laboratory Southern Medical University Affiliated Fengxian Hospital Shanghai China

**Keywords:** angiogenesis, inflammation, migration, nanotube formation, proliferation, TNFAIP2

## Abstract

TNFα‐induced protein 2 (TNFAIP2) is a primary response gene of TNFα. TNFAIP2 is highly expressed in immune cells and the urinary bladder. The expression of TNFAIP2 is regulated by multiple transcription factors and signalling pathways, including NF‐κB, KLF5 and retinoic acid. Physiologically, TNFAIP2 appears to be a multiple functional mediator not only for inflammation, angiogenesis and tunneling nanotube (TNT) formation but also as a regulator of cell proliferation and migration. The expression of TNFAIP2 is frequently abnormal in human cancers and in infectious diseases. Due to its significant functions in cell proliferation, angiogenesis, migration and invasion, TNFAIP2 could be a potential diagnostic biomarker and therapeutic target for cancer.

## 1 INTRODUCTION

Tumour necrosis factor alpha (TNFα) is a proinflammatory cytokine that is mainly secreted by immune cells, including macrophages, lymphocytes and mast cells.^1^ TNFα plays multiple roles in cell proliferation, apoptosis, differentiation, lipid metabolism and inflammation. Accumulating evidence suggests that the dysregulation of TNFα synthesis has been implicated in human autoimmune diseases and tumourigenesis.^2^ TNFα exerts its effect by regulating gene expression and diverse signalling cascades.

In recent years, TNFα‐induced proteins (TNFAIPs) have been well studied. TNFα‐inducible protein 2 (*TNFAIP2*), also named B94 or M‐Sec, was first identified as a primary response gene in TNFα‐treated HUVECs.^3^ TNFAIP2 can be induced by other cytokines, such as IL‐lβ, LPS,^3^, ^4^ interferon‐γ,^4^ serum, PDGF, FGF^5^ and TPA.^6^ *TNFAIP2* is a single copy gene that is evolutionarily conserved.^3^ The predicted amino acid sequence of mouse TNFAIP2 was found to be 83% identical to its human homolog.^5^ TNFAIP2 plays essential roles in inflammation, angiogenesis, migration and nanotube formation.

**Figure 1:**
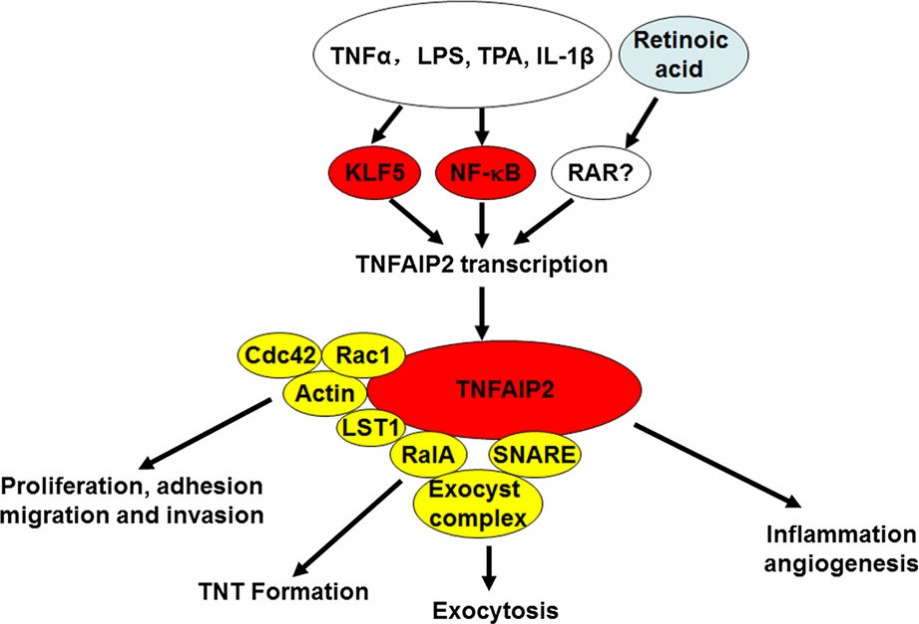
The functions, regulatory mechanisms and interacting proteins for TNFAIP2

In this review, we first summarize the biochemistry of TNFAIP2, including its gene and protein structures, expression patterns, interacting proteins, and signalling pathways. Then, we review the cellular and physiological functions of TNFAIP2, including inflammation, angiogenesis, cell proliferation, migration, membrane nanotube formation and antivirus process (Figure 1). In addition, the abnormal expression of TNFAIP2 in human diseases is summarized. Finally, we discuss the future research directions for TNFAIP2.

## 2 BIOCHEMISTRY

### 2.1 Gene and protein structures

The *TNFAIP2* gene is located at the q32 region of chromosome 14, spanning 11.11 kb genomic DNA with 11 exons. The full‐length cDNA of human *TNFAIP2* consists of 4180 bp with a 131‐bp 5′‐untranslated region (UTR), a 2083‐bp 3′‐UTR and a 1965‐bp sequence coding for a 654‐amino acid polypeptide.^5^

*TNFAIP2* cDNA encodes a 73‐kDa polypeptide, which consists of helix bundles arranged in a straight rod‐like shape, similar to the membrane tethering complex subunits. The crystal structure of the near‐full‐length TNFAIP2 structure has significant similarity to subunits of membrane tethering complexes, including the exocyst complex, the Dsl1 complex, the conserved oligomeric Golgi (COG) complex and the Golgi‐associated retrograde protein (GARP) complex.^7^

### 2.2 Expression

TNFAIP2 is most abundant in immune cells and the urinary bladder at both mRNA and protein levels. Based on Northern blot analysis, *TNFAIP2* mRNA is highly expressed in the spleen, lymph node, fetal kidney, fetal and adult lung, and placenta. The expression of TNFAIP2 is also enriched in endothelial cells,^3^ myelomonocytic cells, peripheral blood monocytes,^5^ intestinal M cell, dendritic cells, macrophages^4^ and mature sperm.^3^ During mouse development, TNFAIP2 follows the course of hematopoiesis and is successively expressed in the fetal liver, adult spleen and bone marrow. While most tissues express a 4.1‐kb transcript of TNFAIP2, a 2.5‐kb transcript is expressed in the mouse placenta and testes.^3^

TNFAIP2 protein is mainly localized to the cytosol and the Golgi apparatus and additionally localized to the nucleus and nuclear membrane. TNFAIP2 is also enriched at the actin‐based membrane ruffles and protrusions^6^, ^8^ and was predicted to be an intracellular protein by several bioinformatic analyses.

### 2.3 Interacting proteins

To date, 6 TNFAIP2 interacting proteins have been identified. The co‐immunoprecipitation assays showed that TNFAIP2 interacts with actin and is involved in the formation of actin‐based membrane protrusions in NPC‐TW02 cells.^4^, ^9^ We reported that TNFAIP2 interacts with the 2 small GTPases, Rac1 and Cdc42, thereby regulating actin cytoskeleton and cell morphology in breast cancer cells. In vitro GST‐pulldown assay indicated that TNFAIP2 directly interacts with Rac1, but not Cdc42.^6^ Similarly, another small GTPase RalA has been shown to directly bind to TNFAIP2 to induce the membrane nanotube formation in HeLa cells.^4^ Schiller et al^10^ found that leucocyte‐specific transcript 1 (LST1) directly interacts with TNFAIP2 to mediate the formation of functional nanotubes. In addition, the Reference Genome Annotation Project predicates that TNFAIP2 is a soluble N‐ethylmaleimide‐sensitive factor attached protein receptor (SNARE)‐binding protein.

### 2.4 Signalling pathways

#### 2.4.1 Nf‐κb

TNFAIP2 expression induced by TNFα depends on the NF‐κB transcription factor. Depletion of the p65 subunit of NF‐κB abolished the TNFα‐induced expression of TNFAIP2. Three NF‐κB binding sites (−2362*, −2708* and −2718 from the ATG start codon) were identified at the upstream region of *TNFAIP2*.^11^ Another independent study validated that TNFAIP2 is a NF‐κB dependent gene.^12^ A microarray analysis in myeloma cells demonstrates that TNFAIP2 is significantly induced by cell adhesion to fibronectin, which is correlated with the activation of NF‐κB.^13^

In nasopharyngeal carcinoma, the latent membrane protein 1 (LMP1) can transcriptionally induce *TNFAIP2* expression via NF‐κB. The study revealed that NF‐κB inhibitor (BAY11‐7082) or depletion of p65 largely reduced the LMP1‐induced TNFAIP2 expression, whereas ectopic expression of p65 is sufficient to induce TNFAIP2 expression. Luciferase reporter assays demonstrated that a newly identified NF‐κB binding site upstream (−3869 to −3860) of the *TNFAIP2* transcription start site is responsible for NF‐κB‐mediated *TNFAIP2* transcription.^8^

In another study, 2 NF‐κB‐binding sites at the *TNFAIP2* gene promoter were identified. The first site is located at 419‐435 base pairs upstream of the transcription start site of *TNFAIP2*. The second site is located at 3994‐4002 base pairs upstream of the transcription start site of *TNFAIP2*. However, the ChIP experiment results indicated that p65 binds to the second site exclusively in a time‐dependent manner. Binding of p65 was accompanied by the recruitment of RNA polymerase II in *L. pneumophila‐*infected A549 cells.^14^

#### 2.4.2 Retinoic acid

Retinoids, the natural and synthetic derivatives of vitamin A, can induce cell differentiation and be used for cancer treatment and prevention. TNFAIP2 could be involved in RA signalling. Two independent microarray studies indicated that *TNFAIP2* is a potential retinoic acid target gene.^15^, ^16^ All‐trans‐retinoic acid (ATRA) strongly upregulates *TNFAIP2* in PML‐RARα‐positive myeloid leukaemia cells or lymphoma cells.

*TNFAIP2* was greatly induced by RA in BEAS‐2B human bronchial epithelial cells.^17^ In RA‐resistant bronchial epithelial cells, RA failed to induce TNFAIP2 expression. These results indicated that TNFAIP2 may be linked to the RA resistance of human bronchial epithelial cells.

RA may be therapeutic for endometrial cancer. TNFAIP2 was induced by the RA agonist AM580 in endometrial cancer cells.^18^ qRT‐PCR verified that AM580 significantly induced TNFAIP2 expression. Furthermore, ChIP assays demonstrated that ligand‐activated RARα binds to the promoter of *TNFAIP2*.

#### 2.4.3 KLF5

We found that *TNFAIP2* was a direct downstream target gene of KLF5. A microarray study in the TSU‐Pr1 bladder cancer cell line indicated that KLF5 induced the expression of *TNFAIP2*.^19^ Then, we demonstrated that KLF5 and TNFAIP2 are coexpressed in breast cell lines and tumours, and KLF5 directly binds to the 2 *Sp1* sites at the *TNFAIP2* gene promoter to regulate its transcription. Moreover, KLF5 promotes breast cancer cell proliferation, migration and invasion, at least in part, through TNFAIP2.^6^

Interestingly, both KLF5 and TNFAIP2 are induced by proinflammatory factors, such as TNFα,^20^ lipopolysaccharide,^21^ IL‐1β,^22^ and TPA.^3^, ^6^ KLF5 has been shown to form a complex with NF‐κB to regulate target genes.^23^ It is likely that KLF5 and NF‐κB form a transcription complex at the *TNFAIP2* gene promoter and coordinately regulate *TNFAIP2* gene transcription.

In addition, KLF5 also interacted with the RAR, and synthetic RAR ligands modulated KLF5 transcriptional activity and affected stress responses in the cardiovascular system in a KLF5‐dependent manner.^24^ Consistently, ATRA inhibited the proliferation of intestinal epithelial cells by inhibiting the expression of KLF5.^25^ Synthetic retinoid Am80 suppresses KLF5 expression and smooth muscle phenotypic modulation and in‐stent neointima formation.^26^ Thus, the induction of TNFAIP2 by RA may be cell line‐specific and KLF5‐independent.

Finally, a ChIP‐seq analysis in HEK293 cells indicated that *TNFAIP2* is one of 59 target genes of BTB and CNC homology 1 (BACH1).^27^ BACH1 constitutes a major link between the cellular heme level, the redox state and the transcriptional response. The function of TNFAIP2 in the heme‐BACH1 pathway needs further study.

## 3 CELLULAR AND PHYSIOLOGICAL FUNCTIONS OF TNFAIP2

### 3.1 Inflammation

TNFAIP2 is involved in the NF‐κB signalling pathway to regulate the cell inflammatory response. TNFAIP2 is phosphorylated by PLK upon LPS stimulation.^28^ The *TNFAIP2* gene promoter is highly acetylated at histone H4 in *L. pneumophila‐*infected human blood‐derived macrophages (BDMs). Furthermore, TNFAIP2 is highly induced at both the mRNA and protein levels after infection with *L. pneumophila* in lung epithelial cells and myeloid cells. Knockdown of TNFAIP2 suppressed the intracellular growth of *L. pneumophila*.^14^ Interestingly, TNFAIP2 has been associated with rheumatoid arthritis and autoimmune myocarditis in genome‐wide association studies (Wellcome Trust Case Control Consortium, 2007; Kuan et al 1999). A recent study showed that miR‐221 alleviates the inflammatory response and cell apoptosis of neuronal cell through targeting TNFAIP2 in spinal cord ischemia‐reperfusion.^29^ Oxygen‐glucose deprivation induced the expression of TNFAIP2 mRNA and protein in neuron cells and TNFAIP2 is a direct target gene for miR‐221.^29^

### 3.2 Angiogenesis

TNFAIP2 is an important angiogenic factor. In an in vitro angiogenesis experiment, the HUVECs could form vascular‐like structures within 1 to 2 hours of plating onto the matrigel. TNFAIP2 expression increased with the formation of capillary tube‐like structures.^3^ During mouse embryogenesis, TNFAIP2 expression patterns followed the course of hematopoiesis. It was successively expressed in the myocardium and liver. After birth, its expression shifted to the spleen and thymic medulla.^5^ Based on the immunohistochemical staining in 95 nasopharyngeal carcinoma biopsy specimens, a significant correlation between TNFAIP2 expression and intratumoral microvessel density was observed.^30^

TNFAIP2 is a novel inflammatory regulator of chemokine secretion from endothelial cells that facilitates T cell transendothelial migration. The silencing of TNFAIP2 in activated endothelial cells decreased the transendothelial migration of effector T lymphocytes by reducing the preferential secretion of endothelial‐produced CCL2, IL‐6 and GM‐CSF.^31^

### 3.3 Cell proliferation

We reported that KLF5 induces TNFAIP2 to promote triple‐negative breast cancer cell proliferation. In a xenograft mouse model, stable knockdown of TNFAIP2 in HCC1937 cells significantly suppressed tumour growth and reduced the tumour weight in NOD‐SCID mice.^6^ Furthermore, Xie et al reported that the inhibition of TNFAIP2 expression leads to a decreased rate of proliferation and a significant reduction in colony formation in oesophageal squamous cell carcinoma. TNFAIP2 knockdown in oesophageal squamous cell carcinoma cells arrested the cells in the G0/G1 phase.^32^

### 3.4 Adhesion and migration

We found that TNFAIP2 contributes to KLF5‐induced cell adhesion, migration and invasion in breast cancer.^6^ TNFAIP2 interacts with the 2 small GTPases Rac1 and Cdc42 and regulates the formation of filopodia and lamellipodia, which provide force to cell motility.^6^

Chen et al reported that TNFAIP2 contributes to LMP1‐induced cell motility in nasopharyngeal carcinoma. TNFAIP2 is associated with actin, modulates actin‐based protrusion formation and promotes the migration of nasopharyngeal carcinoma cells.^8^ In agreement with this, the TNFAIP2 expression was significantly correlated with distant metastasis‐free survival in nasopharyngeal carcinoma patients. In the TNFAIP2 high expression nasopharyngeal carcinoma specimen group, 40.5% of patients developed distant metastasis; this proportion was only 12.1% in the TNFAIP2 low expression group. Knockdown of TNFAIP2 in nasopharyngeal carcinoma HK1 cells dramatically reduced cell migration and invasion but had no significant impact on cell growth.^30^

Additionally, TNFAIP2 was found to promote cell migration and invasion via the activation of the Wnt/β‐catenin signalling pathway in oesophageal squamous cell carcinoma.^32^ Knockdown of TNFAIP2 inhibits the expression of β‐catenin and its downstream targets, including C‐Myc, Cyclin D1, MMP‐7 and Snail, and upregulates the expression of E‐cadherin and p‐GSK‐3β.

### 3.5 Membrane nanotube formation

Tunneling nanotube (TNT) is a new type of cell‐cell communication characterized as thin and long membranous protrusions connecting remote cells. TNT structures mediate the intercellular transport of various components, including calcium, proteins, organelles and HIV virus, in a variety of cell types, such as B cells,^33^ T cells,^34^ macrophages,^35^ mast cells,^36^ NK cells^37^ and dendritic cells.^38^

TNFAIP2 is recognized as a TNT marker and central factor for TNT formation.^39^ Depletion of TNFAIP2 drastically reduces endogenous TNT formation as well as intercellular calcium flux in Raw264.7 macrophages**.** The transient overexpression of TNFAIP2 in HeLa cells induces close‐ended TNT structures only when TNFAIP2 is stably expressed. Furthermore, TNFAIP2 coordinates with RalA small GTPase and the exocyst complex to remodel the actin cytoskeleton and initiate the formation of membrane nanotubes.^4^, ^40^

Further study indicated that the N‐terminal polybasic region of TNFAIP2 directly binds phosphatidylinositol (4,5)‐bisphosphate for its localization to the plasma membrane during the initial stage of TNT formation, and a positively charged surface in the C‐terminal domains is responsible for TNFAIP2 interaction with active RalA.^7^

Christian et al reported that the transmembrane MHC class III protein LST1 can serve as a scaffold and induce TNT formation by cooperating with TNFAIP2, RalA and the exocyst complex.^10^

TNFAIP2 was implicated in the TNT development, which depends on p53 activation in astrocytes.^41^ The activation of p53 with H_2_O_2_ or serum deprivation treatments significantly increases *TNFAIP2* expression at the mRNA level. The overexpression of the p53‐dominant negative mutant abolished the upregulated expression of TNFAIP2 in the H_2_O_2_ treatment group, but not in the serum deprivation treatment group. These results suggest that p53 activation induces TNFAIP2 expression, which collaborates with RalA and the exocyst complex to promote TNT formation in the stressed cells.

## 4 GENETIC AND EXPRESSION ALTERATIONS OF TNFAIP2 IN CANCERS AND INFECTIOUS DISEASES

### 4.1 Genetic alterations

Single‐nucleotide polymorphisms (SNP) at the 3′UTR of *TNFAIP2* have been linked to several diseases. In squamous cell carcinoma of the head and neck, the rs8126 variant C allele greatly reduced luciferase activity and mRNA expression of *TNFAIP2* and increased cancer risk in an allele dose‐response manner compared with the rs8126 TT genotype.^9^ In another study, the rs8126 CC genotype was significantly associated with an increased risk of gastric cancer compared with the combined rs8126 TT+TC genotypes.^42^ In normal oesophagus tissues, carriers of the rs8126 CC and CT genotypes had significantly lower *TNFAIP2* mRNA levels than those with the TT genotypes.^43^ Furthermore, the SNP at the 3′ UTR of TNFAIP2 (rs8126 T > C) is the binding site of miR‐184. miR‐184 is inversely correlated with *TNFAIP2* mRNA and protein expression levels in glioma.^44^ In septic shock patients, the 3′UTR SNP (rs8126) of *TNFAIP2* is associated with the higher mortality of septic shock patients. Compared with the A allele, the G allele of *TNFAIP2* rs8126 enhanced TNFAIP2 expression, decreased IL‐8 production, reduced the survival and increased organ dysfunction in patients experiencing septic shock.^45^

### 4.2 Expression aberrations

Expression of TNFAIP2 was found to be abnormal in cancers, bacteria and virus infectious diseases. In different types of cancer, TNFAIP2 has been suggested to be an oncogene based on its positive role in cell proliferation, angiogenesis and migration.

Chen et al reported that TNFAIP2 is one of 10 most highly induced genes in nasopharyngeal carcinoma tissues compared with the adjacent normal tissues. TNFAIP2 is highly expressed in nasopharyngeal carcinoma tissues, and increased TNFAIP2 expression is significantly correlated with low distant metastasis‐free survival in patients.^30^ In breast cancer, the expression of TNFAIP2 is significantly increased in triple‐negative breast cancer (TNBC) samples compared with normal tissues based on the analysis of the TCGA (The Cancer Genome Atlas) database.^6^ Cheng et al reported that TNFAIP2 expression is elevated in glioma tissues compared with normal brain tissues, and the expression is higher in high‐grade gliomas (WHO grades III and IV) than in low‐grade gliomas.^44^ In addition, TNFAIP2 is overexpressed in oesophageal squamous cell carcinoma.^32^ Furthermore, TNFAIP2 expression is significantly associated with tumour grade in oesophageal squamous cell carcinoma patients, and high levels of TNFAIP2 expression indicate shorter disease‐free survival.^32^

**Figure 2:**
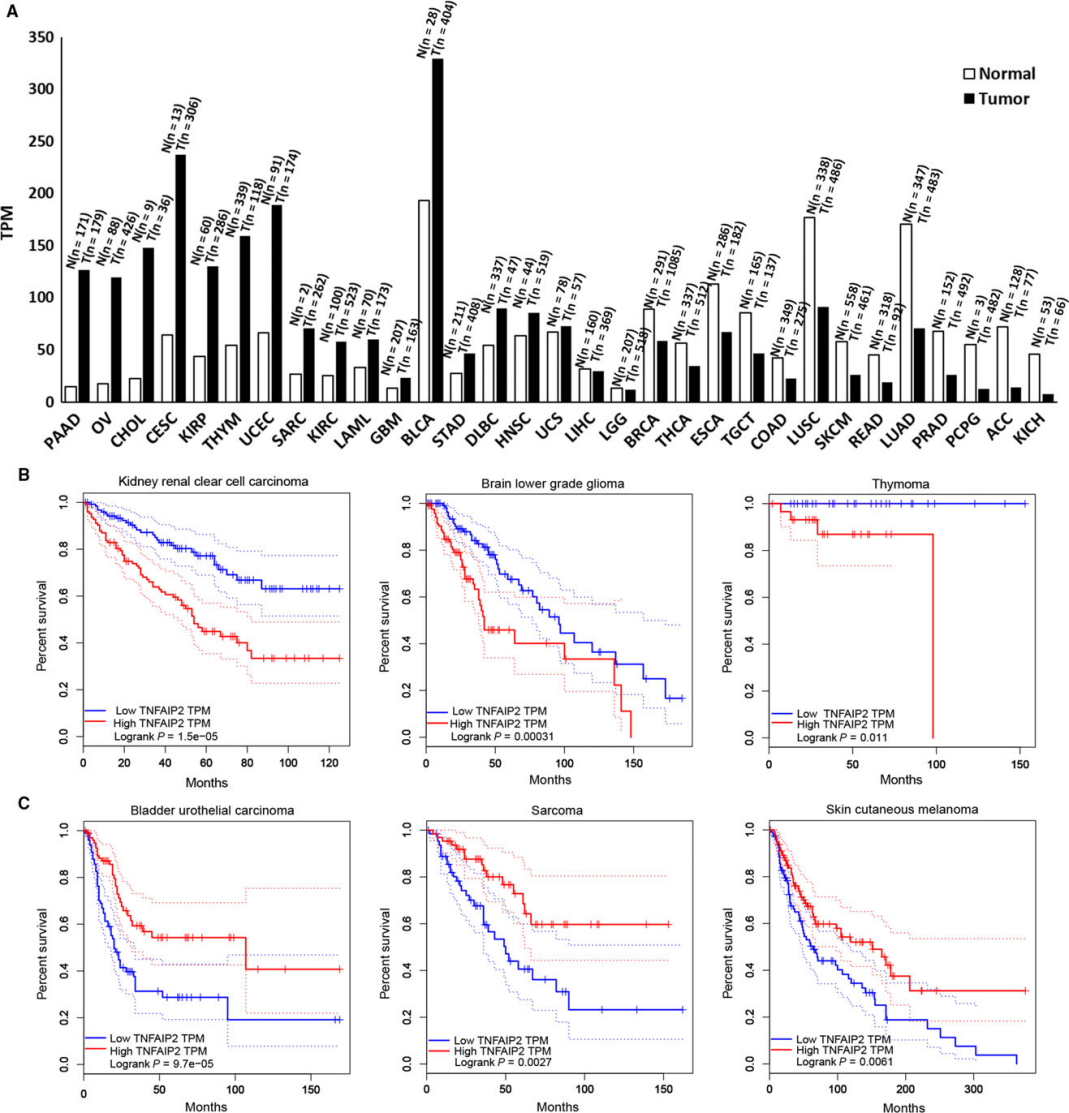
The expression alterations of TNFAIP2 and its prognosis values in different cancers. A, *TNFAIP2* mRNA expression levels in 31 cancers. The height of bar represents the median expression levels of certain tumour types and normal tissues. The tumour and normal numbers are listed on top of each bar. *TNFAIP2* mRNA levels are upregulated in different cancers, including PAAD (Pancreatic Adenocarcinoma), OV (Ovarian Serous Cystadenocarcinoma), CHOL (Cholangio Carcinoma), CESC (Cervical Squamous Cell Carcinoma and Endocervical adenocarcinoma), KIRP (Kidney Renal Papillary Cell Carcinoma), THYM (Thymoma), UCEC (Uterine Corpus Endometrial Carcinoma), SARC (Sarcoma), KIRC (Kidney Renal Clear Cell Carcinoma), LAML (Acute Myeloid Leukemia), GBM (Glioblastoma Multiforme), BLCA (Bladder Urothelial Carcinoma), STAD (Stomach Adenocarcinoma), DLBC (Lymphoid Neoplasm Diffuse Large B‐cell Lymphoma) for more than 1.5 fold. TNFAIP2 mRNA levels are not changed in HNSC (Head and Neck Squamous Cell Carcinoma), UCS (Uterine Carcinosarcoma), LIHC (Liver Hepatocellular Carcinoma) and LGG (Brain Lower Grade Glioma). TNFAIP2 mRNA levels are downregulated in different cancers, including BRCA (Breast Invasive Carcinoma), THCA (Thyroid Carcinoma), ESCA (Esophageal Carcinoma), TGCT (Testicular Germ Cell Tumors), COAD (Colon Adenocarcinoma), LUSC (Lung Squamous Cell Carcinoma), SKCM (Skin Cutaneous Melanoma), READ (Rectum Adenocarcinoma), LUAD (Lung Adenocarcinoma), PRAD (Prostate Adenocarcinoma), PCPG (Pheochromocytoma and Paraganglioma), ACC (Adrenocortical Carcinoma) and KICH (Kidney Chromophobe) for more than 1.5 fold. B, A high *TNFAIP2* mRNA level is significantly associated with a short survival in several cancer types, including kidney renal clear cell carcinoma, brain lower grade glioma and thymoma. The y axis represents survival rate and the x axis represents alive time (month) of patients. C, A high *TNFAIP2* mRNA level is significantly associated with a long survival in several cancers, including bladder urothelial carcinoma, sarcoma and skin cutaneous melanoma

We analysed mRNA expression variations of TNFAIP2 in more than 30 cancer types in the TCGA database using an online tool GEPIA (Gene Expression Profiling Interactive Analysis, http://gepia.cancer-pku.cn/index.html). As shown in Figure 2A, the *TNFAIP2* mRNA expression levels are upregulated in PAAD (Pancreatic Adenocarcinoma), OV (Ovarian Serous Cystadenocarcinoma), CHOL (Cholangio Carcinoma), CESC (Cervical Squamous Cell Carcinoma and Endocervical adenocarcinoma), KIRP (Kidney Renal Papillary Cell Carcinoma), THYM (Thymoma), UCEC (Uterine Corpus Endometrial Carcinoma), etc. In contrast, it is downregulated in KICH (Kidney Chromophobe), ACC (Adrenocortical Carcinoma), PCPG (Pheochromocytoma and Paraganglioma), PRAD (Prostate Adenocarcinoma), LUAD (Lung Adenocarcinoma), etc.

Besides the mRNA expression analysis, we also investigated diagnostic and prognostic values of *TNFAIP2*. A high *TNFAIP2* mRNA level is significantly associated with a short survival in several cancer types, including kidney renal clear cell carcinoma, brain lower grade glioma and thymoma (Figure 2B). On the contrary, a high *TNFAIP2* mRNA level is significantly associated with a long survival in several cancers, including bladder urothelial carcinoma, sarcoma and skin cutaneous melanoma (Figure 2C). These results imply that TNFAIP2 could be a potential diagnostic biomarker and therapeutic target for cancers.

TNFAIP2 is highly expressed in normal marrow and in marrow from patients with acute myelogenous leukaemia (French‐American‐British subtypes M0‐M2) but is suppressed in marrow cells from APL patients.^15^ Kondratiev et al reported that TNFAIP2 is strongly expressed in classical Hodgkin lymphoma, nodular lymphocyte predominant Hodgkin lymphoma and primary mediastinal (thymic) large B cell lymphoma compared with diffuse large B cell lymphoma, Burkitt's lymphoma and anaplastic large cell lymphoma, suggesting that TNFAIP2 is a sensitive and specific marker for Hodgkin lymphoma and primary mediastinal (thymic) large B cell lymphoma.^46^

## 5 CONCLUSIONS

In summary, *TNFAIP2* is a primary response gene induced by multiple proinflammatory molecules at the level of transcriptional activation. The transcription of *TNFAIP2* is regulated by different transcription factors, including NF‐κB, KLF5 and RAR. TNFAIP2 has important functions in different cellular and physiologically processes, including cell proliferation, adhesion, migration, membrane TNT formation, angiogenesis, inflammation and tumourigenesis. The expression of TNFAIP2 is frequently altered in human diseases, including cancers and infectious diseases.

## 6 PERSPECTIVES

Although some of the biochemical and cellular characteristics of TNFAIP2 have been studied in the last 2 decades, as reviewed above, the roles of TNFAIP2 under physiological and pathological conditions are still far from clear. The posttranslational modifications, interacting proteins, transcriptional regulation and downstream functional mechanisms of TNFAIP2 and whether TNFAIP2 can be developed as a diagnostic and therapeutic target require further investigation.

TNFAIP2 regulates the actin cytoskeleton, especially the membrane protrusions, to promote cell migration and invasion by regulating the activity of the small GTPase Cdc42 and Rac1 in breast cancer and nasopharyngeal carcinoma. TNFAIP2 also interacts with another GTPase RalA and the exocyst complex to induce TNT formation. Both the protrusions and TNT are membrane structures based on actin polymerization. However, whether TNFAIP2‐induced actin‐rich protrusions and TNT share similar molecular mechanisms is still unclear. How TNFAIP2 interacts with actin and regulates actin assembly and affects cell morphology and mobility warrants further investigation.

Since TNFAIP2 plays an important role in cell migration and invasion in breast cancer, nasopharyngeal carcinoma and oesophageal squamous cell carcinoma, it would be significant to further determine whether TNFAIP2 promotes metastasis in a mouse xenograft tumour model. It is also important to develop inducible and tissue‐specific *TNFAIP2* transgenic animal models to study the physiological and pathological functions of TNFAIP2 in normal organ development and diseases. To this end, we developed a *Tnfaip2* flox mouse model.

TNFAIP2 has potential as a biomarker for diagnosis and prognosis in a variety of cancers and infectious diseases. The expression of TNFAIP2 is significantly correlated with low distant metastasis‐free survival in patients with nasopharyngeal carcinoma, and high levels of TNFAIP2 expression indicate shorter disease‐free survival in oesophageal squamous cell carcinoma patients. Therefore, it is necessary to develop reagents and methods, especially standard IHC techniques, to accurately and conveniently measure changes in TNFAIP2 protein expression in cancer specimens.

Small molecular inhibitors, anti‐*TNFAIP2* siRNAs, or monoclonal antibodies of TNFAIP2 might develop into anti‐cancer drugs. On the other hand, the small molecular inhibitors or monoclonal antibodies targeting the positive upstream regulators or downstream target proteins of TNFAIP2 could be another choice for disease treatment.

## CONFLICT OF INTEREST

The authors declare that they have no conflict of interest.
